# Are national suicide prevention programs effective? A comparison of 4 verum and 4 control countries over 30 years

**DOI:** 10.1186/s12888-019-2147-y

**Published:** 2019-05-23

**Authors:** U. Lewitzka, C. Sauer, M. Bauer, W. Felber

**Affiliations:** Department of Psychiatry and Psychotherapy, University Hospital Carl Gustav Carus, Technische Universität Dresden, Fetscherstr. 74, D-01307 Dresden, Germany

**Keywords:** Suicide, Suicide prevention programs, Suicide rates, Effectiveness

## Abstract

**Background:**

Suicide and non-fatal suicidal behavior are significant public health issues worldwide requiring effective preventive interventions.

**Methods:**

The aim of the present study was to analyze the effectiveness of national suicide prevention programs taking a statistical approach involving the segmented regression analysis of interrupted time series data.

**Results:**

This study demonstrates that National Suicide Prevention Programs are effective, but this effect seems to correlate with age and sex. Our data have shown a statistical significant decline in suicide rates in the verum countries in males, with the strongest effects in groups aged 25-to-44 years and 45-to-64 years.

**Conclusion:**

Our study implies that the implementation of a national strategy is an effective tool to reduce suicide rates.

## Background

### Epidemiology

The World Health Organisation (WHO) estimates that about a million people die by suicide every year, representing a “global” mortality rate of 16 per 100,000 or one death every 40 s making it the tenth leading cause of death worldwide [1]. Suicide rates in many developing countries have been steadily rising, and the overall worldwide suicide rate has increased during the last 50 years [2].

Suicide and non-fatal suicidal behavior are significant public health issues worldwide requiring effective preventive interventions. However, there is still a need to identify what prevention strategies should be prioritized to achieve the biggest impact on a reduction of suicide attempts and suicide deaths [3].

Suicidality is a problem caused by multiple factors, making it difficult to treat by individual medical, psychological, educational, social or political methods.

Thus national suicide prevention programs (NSPP) were initiated in the 1990s aiming to take a holistic approach to combat suicide. There are 28 countries known to have national strategies for suicide prevention. Prevention programs are designed to identify vulnerable groups, improve the assessment and care of people with suicidal behavior, and improve surveillance and research. They also aim to raise awareness by improving public education. NSPPs attempt counter the stigma toward people exhibiting suicidal behavior and those who suffer from mental disorders. Institutions such as the World Health Organization (WHO) and the International Association for Suicide Prevention (IASP) have developed common guidelines and the following recommendations to set up suicide prevention strategies including NSPP [2, 4, 5]:

1. Preventive measures should address suicide and suicide attempts. The loss of human resources, socioeconomic burden, and costs for the healthcare of these individuals are considerable.
2. The support and rehabilitation of persons at risk can prevent some suicides. A holistic approach is necessary.
3. National governments are responsible for developing strategies to provide financial and technical support that involve society as a whole.
4. Measurable objectives and systematic studies must be forthcoming.

### National suicide prevention programs in Norway, Sweden, Finland and Australia

Norway published the first national suicide prevention strategy in 1995, one that has been revised and updated several times. Aspects of the second and third prevention strategies are the focus of their program. Approaches to enhance the mindfulness of politicians, governmental departments and the general population were taken for this purpose. Medical and social welfare programs were optimized, and aftercare improved. An external board was assigned to evaluate individual projects and the entire program [5, 6]. Their findings were summarized in a publication by Sørås in 2000 [7]. A follow-up project to the national plan “Measures against suicide 2000-2002” was evaluated and published by Mehlum and Reinholdt in 2001 [8].

The aim of Sweden’s national suicide prevention program established in 1995 was a consistent drop in the number of suicides and suicide attempts, to reduce the factors encouraging suicidal behavior in children and youths, as well as the early detection of suicidal tendencies in endangered groups. They aspired to increase the level of awareness in the general population in the first, second and third prevention strategies [9].

From 1986 to 1991, Finland enacted a research program on suicide and developed preventive strategies. One major goal was to engage high-risk groups. A suicide prevention program was implemented in 1992 as the first governmental program with activities involving all levels of prevention strategies [10]. Moreover, an external board was assigned to evaluate and improve this program [11].

A national youth-suicide prevention program existed from 1995 to 1999 in Australia. This initiative then evolved into a national suicide prevention program involving first, second, and third prevention strategies. It was one of this program’s aims to observe a lower suicide rate and reduction in suicidal thinking and behavior. The program also intended a better psychological strain and mental health [12]. Following implementation of the original National Youth Suicide Prevention Strategy (NYSPS) in 1995, they did in fact observe a substantial decline in suicide in young men. A study by Page et al. [13] reported a minor discernible impact on suicide rates in those areas that had participated in local targeted suicide prevention activities in the period following the NYSPS.

One of our main problems is how to evaluate suicide prevention programs. As Kerkhof and Clark [14] stated in their editorial, there are obvious limitations in studying effectiveness, e.g., there are no experimental designs that might be applied. So far, little research has been done investigating the effect of national suicide prevention programs, whereas there have been studies about local interventions programs to prevent suicide and suicide attempts; e.g. [15].

The aim of the present study was to analyze the effectiveness of national suicide prevention programs taking a statistical approach involving the segmented regression analysis of interrupted time series data. We posed the following questions:

1. Does the implementation of a national suicide prevention program lead to a significant reduction in suicide rates?
2. Are there gender-related differences?
3. Are there age-related differences?

## Methods

One of the major difficulties is verifying the success of NSPP, which we considered as a decrease in suicide attempt or suicide death rates within the population of a country. It is extremely hard to tell which parameters are actually responsible for success among the specific suicide-decreasing effects, spontaneous changes in long-term development, social changes and data inaccessibility due to privacy protection laws, as well as obtaining comparable data. All these factors make it difficult to prove the exact cause of a measured change, making it important therefore to clearly define the structure and approach of the analysis. For the purpose of a conscientious decision-making process three experienced psychiatrists/suicidologists (WF, HT, UL), as well as a statistician (CS), reviewed the existing literature as well as different statistical approaches. Within several face to face meetings, the described approach was defined, and the criteria for the selection of these programs were agreed.

### Criteria for selection of verum and control countries

#### The verum countries

Our main criterion for selecting the verum countries was the existence of a comprehensive national suicide prevention program for at least five years.

Comprehensive statistical analysis should be available. Nations that have an NSPP are Australia, New Zealand, Finland, Norway, and Sweden. As the New Zealanders implemented their program in 1998 only for young people, we had to exclude them from our statistical analyses. The Netherlands, Great Britain, the USA, France, and Estonia have also implemented programs; they failed, however, to meet our inclusion criteria. We ultimately selected Finland, Norway, Sweden, and Australia for the verum group. All these countries have published their programs’ results comprehensively [6–13].

#### Control countries

We compared the verum countries with control countries selected according to the criteria below. They should not have an NSPP and should not differ in the following aspects (at the time of the study):

1. Culture and religion: e.g., suicide rates in Mediterranean countries are lower than in Northern European countries. Other examples are countries with a mostly Muslim population, which may be caused by the strong taboo about this problem in the society.
2. Historical and political factors: studies have shown that substantial historical developments such as political changes have influenced the dynamics of suicide statistics. Thus Eastern European countries, as well as Germany, could not be included.
3. Socio-economic structure: we decided that the control countries should have a western democracy with distinctive market-based economies similar to the verum countries.
4. Population size: Statistics from countries with small populations were not included because lower numbers of suicides, minimal increases or decreases can skew the statistics.
5. Quality of published statistics: data had to be well founded, reliable and accessible, and should have been collected during the same time period. Countries whose statistics were erratically collected were excluded (i.e., African states, China).

Finally, Canada, Austria, Switzerland and Denmark were selected as control countries.

### Criteria for selecting the time period

The verum countries implemented their programs in the 1990s. Long periods of observation were planned due to annual variability, which exerts strong effects, especially in countries with a smaller population. The second reason is the possibility that prevention programs gradually lead to success.

For this study, we had to assess the suicide rates prior to the NSPP to detect any differences. For the verum countries, time 0 (T0) was defined as the year the NSPP was implemented. Countries differed in this respect, thus T0 for Finland is 1992, and 1994 for Norway. T0 is the year 1995 for the control countries. We decided on six years (T0 to T + 5) after the implementation of the NSPP to qualify as the period of analysis. All countries had to be statistically represented at the beginning of the analysis. For that reason, T-22 was established retrospectively as the starting point.

Data were collected from World Health Organization statistics on suicides and demographics separated by age and sex [16].

### Statistics

To estimate the impact of the NSPP on suicide rates in verum and control countries we applied a segmented regression analysis of interrupted time series data [17–21]. This method estimates changes in levels and trends controlling for baseline levels and trends, which is one of its major strengths. The observation period is divided into pre- and post-intervention segments for which separate intercepts and slopes are estimated. A linear relationship between time and outcome is assumed, and a least squares regression line is fitted to each segment of the independent variable. To take into account the autocorrelation among observations, we estimated the effect of the intervention using the ARIMA model (autoregressive integrated moving average) and tested for autocorrelation of the error terms via the Ljung-Box-test.

The time series regression equation for our analysis is:

$$ {\mathrm{Y}}_{\mathrm{t}}={\upbeta}_0+{\upbeta}_1\ast \mathrm{time}+{\upbeta}_2\ast \mathrm{phase}+{\upbeta}_3\ast \mathrm{time}\_\mathrm{after}\_\mathrm{NSPP}+{\mathrm{e}}_{\mathrm{t}} $$

Y_t_ is the outcome variable, in our model this is the number of suicides per 100.000 in year t, “time” is the number of years at time t from the start of the observation period starting with 1 at time point “t-22”; “phase” is an indicator variable, which is 0 for the time points before and 1 for the time points after the NSPP introduction, “time after NSPP” is how many years after NSPP introduction which is set to 0 for the years before the NSPP introduction and taking on the values of 1 to 5 for the years after NSPP introduction and e_t_ represents random variability at time t not explained by the model.

The coefficient β_0_ estimates the baseline level of suicides per 100.000, β_1_ estimates the baseline trend before NSPP introduction, which is the change in the mean number of suicides per 100.000 occurring each year before the implementation. The coefficient β_2_ estimates the change in level in the mean yearly number of suicides per 100.000 immediately after the NSPP implementation and β_3_ estimates the change in trend in the mean number of suicides per 100.000 after its implementation.

All analyses were conducted separately for men and women and split into four age groups each (< 24 years, 25–44 years, 45–64 years and > 65 years). Furthermore, we analyzed the difference in suicide rates between verum- and control countries, again separately for men and women and the different age groups to estimate how the change in suicide rate in the verum countries differed from the change in the control countries. For all analyses, we used SPSS for Windows version 23. A significance level of ≤0.05 was considered significant.

## Results

### Analysis of verum countries - males

**Fig. 1:**
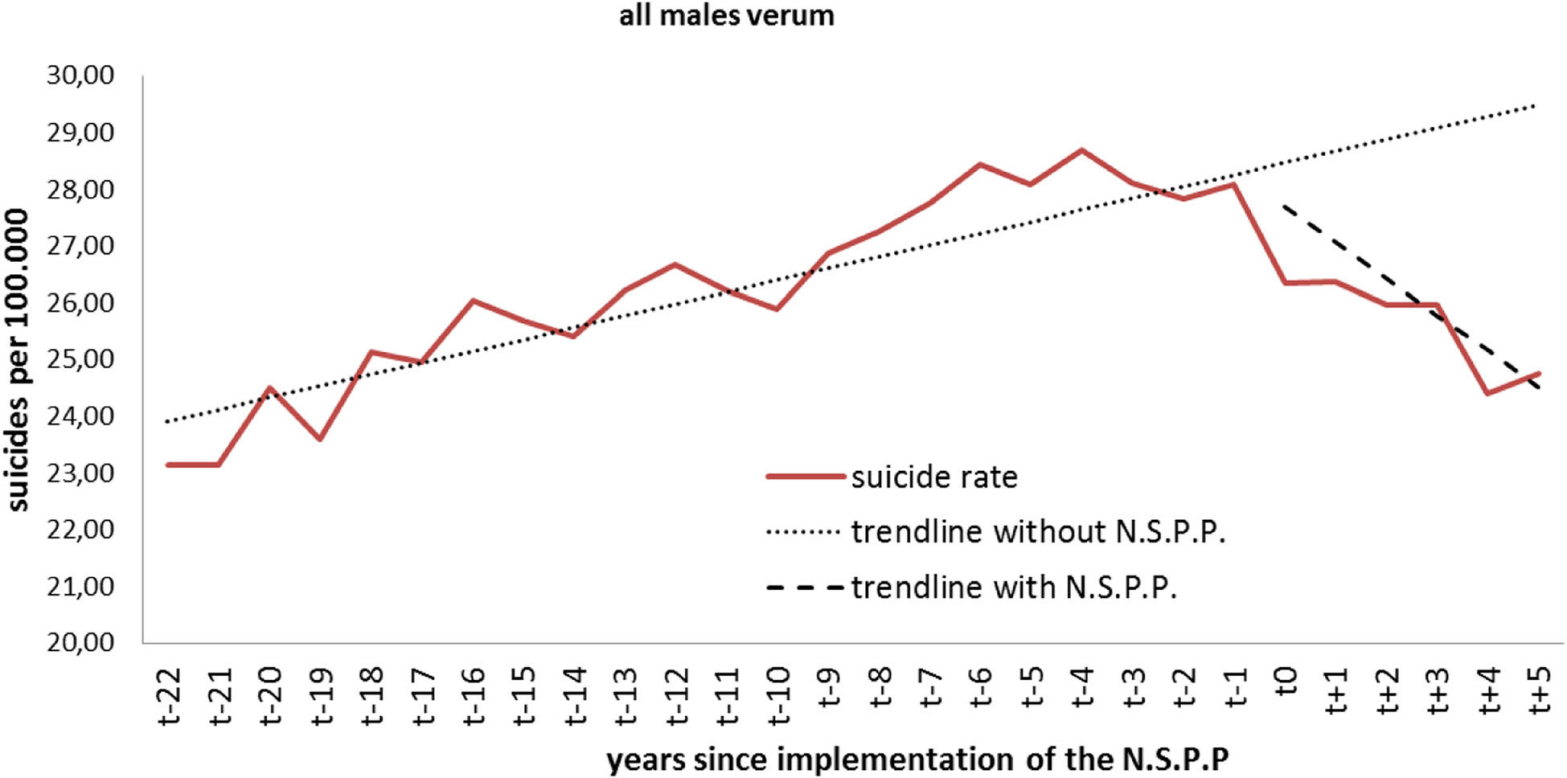
Level change in all males of the verum group

Table 1 and Fig. 1 show the parameter estimates from the linear segmented regression model for all males in the verum countries. Right before the beginning of the observation period, an average of 23 per 100.000 males in the verum countries committed suicide per year. Before implementation of the NSPP, there was a significant year-to-year change in the mean number of suicides (*p* < 0.001). The immediate change in the number of suicides directly after the intervention was not significant (*p* = 0.536). However, the level change becomes significant two years after the intervention and remained significant for the following three years. The year-to-year trend in the mean number of suicides per 100.000 after the intervention changed significantly (*p* = 0.006).

**Table 1:** Verum countries: level and trend change for all males

Verum males all	Coefficient	Standard error	T-statistic	*p*-value
intercept β0	23,705	0,491	48,250	< 0.001
baseline trend β1	0,207	0,038	5437	< 0.001
level change after NSPP β2	−0,764	1216	-0,629	0.536
effect after 1 year	− 1608	1048	− 1534	0.139
effect after 2 years	− 2451	0,935	− 2623	**0.015**
effect after 3 years	− 3295	0,895	− 3680	**0.001**
effect after 4 years	− 4139	0,940	− 4403	**< 0.001**
effect after 5 years	− 4982	1057	− 4711	**< 0.001**
trend change after NSPP β3	−0,843	0,277	− 3045	**0.006**

Observing the different age groups, we detected significant level changes in the group of males under age 24 after 5 years (*p* = 0.049) of NSPP Within the 25-to-44-year-olds, we noted significant level changes after 1 (*p* = 0.041), 2 (*p* = 0.001), 3 (*p* < 0.001), 4 (*p* < 0.001) and 5 (*p* < 0.001) years after the NSPP implementation. The trend change that occurred after the NSPP was implemented also reached significance (*p* = 0.014) in this age group.

The group of 45–64-year-old males revealed significant level changes after 2 (*p* = 0.010), 3 (*p* = 0.001), 4 (*p* = 0.001) and 5 (*p* = 0.001) years of NSPP.

Males older than 65 years showed significant changes in suicide rates after 3 (*p* = 0.011), 4 (*p* = 0.005) and 5 (*p* = 0.007) years of NSPP.

We observed a significant baseline trend in all age groups except that of the males older than 65. These trends were positive for the males younger than 24 years and the group of 25–44-year-olds, which evolved into a negative trend after the NSPP implementation in both groups. However, this trend change only attained significance for the group of the 25-to-45-year-olds (*p* = 0.014). The trend for the group of the 45–64-year-old males was already slightly negative before the NSPP and was strengthened by it, an improvement that did not reach significance (*p* = 0.155).

### Analysis of verum countries - females

**Fig. 2:**
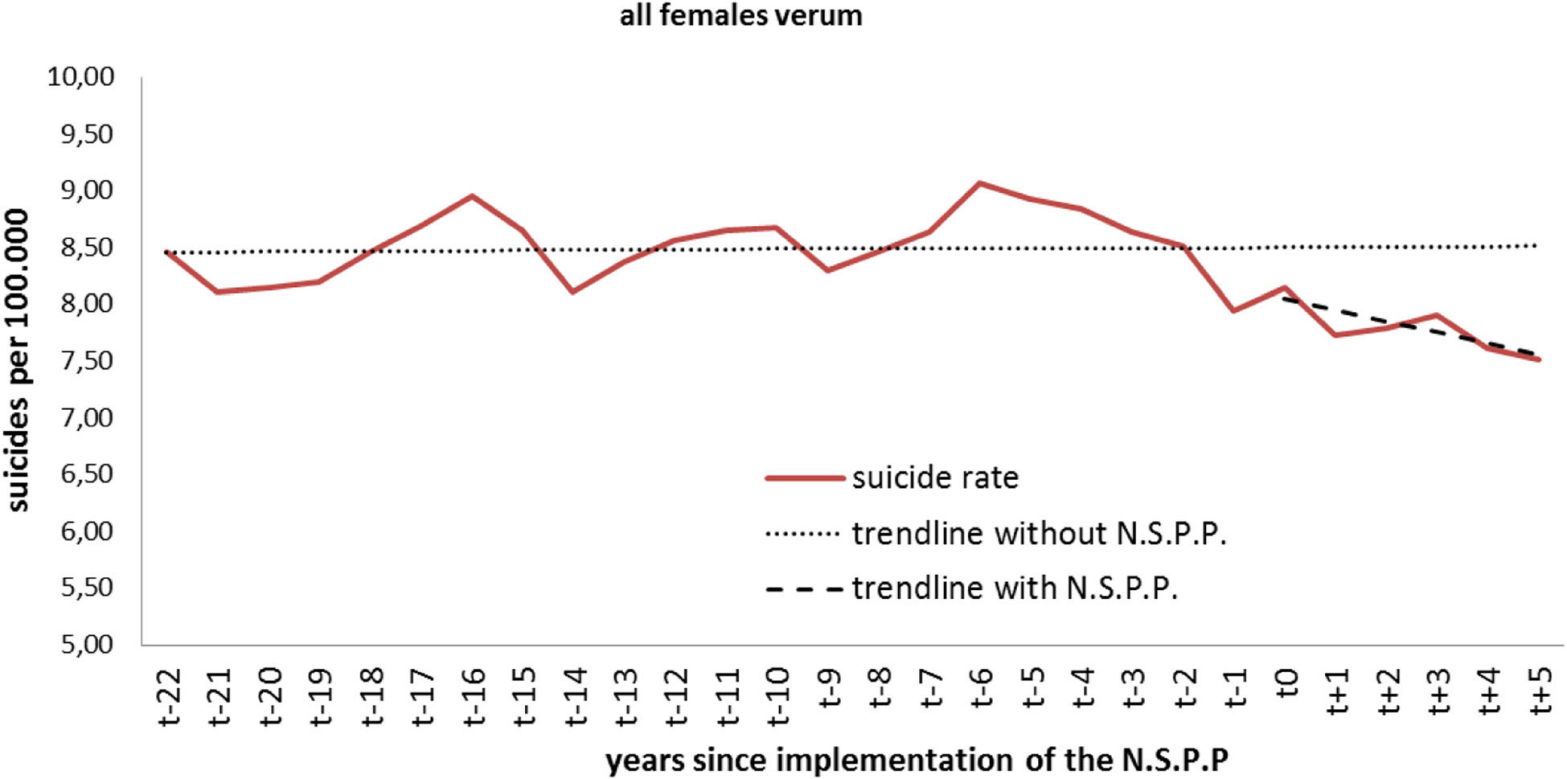
Level change in all females of the verum group

Right before the beginning of the observation period, an average 8 of 100.000 women committed suicide per year in the verum countries. The baseline trend before implementation of the NSPP indicates that the suicide rates remained constant over the years up to the year of NSPP introduction. However, two years after its implementation our analysis showed a statistically significant level change in the suicide rate (*p* = 0.018). The same applies for years 3, 4 and 5 after implementation (Table 2, Fig. 2). The year-to-year trend in the mean number of suicides per 100.000 after implementation did not change significantly (*p* = 0.333).

**Table 2:** Verum countries: level and trend change for all females

All verum females	Coefficient	Standard error	T-statistic	p-value
intercept β0	8460	0,209	40,564	< 0.001
baseline trend β1	0,002	0,015	0,160	0.874
level change after NSPP β2	−0,458	0,332	− 1381	0.325
effect after 1 year	−0,557	0,280	− 1989	0.059
effect after 2 years	−0,655	0,258	− 2540	**0.018**
effect after 3 years	−0,754	0,273	− 2759	**0.011**
effect after 4 years	−0,853	0,321	− 2661	**0.014**
effect after 5 years	−0,951	0,388	−2451	**0.022**
trend change after NSPP β3	−0,099	0,100	−0,989	0.333

Assessing the different age groups, we identified significant level changes in the group of females aged between 45 and 64 years after 3 (*p* < 0.021), 4 (*p* < 0.011) and 5 (*p* < 0.012) years after the NSPP implementation.

Females older than 65 years showed significant level changes immediately after NSPP implementation (*p* = 0.002) and after 1 (*p* < 0.001), 2 (*p* < 0.001), 3 (*p* < 0.001), 4 (*p* < 0.001) and 5 (*p* < 0.001) years of NSPP These two female groups’ baseline trends were significant. The trend was already negative before the NSPP in the group of 45-to-64-year-old females, while the trend for the females older than 65 was constant. The trend became negative in both groups, but the changes did not reach significance.

### Analysis of control countries - males

**Fig. 3:**
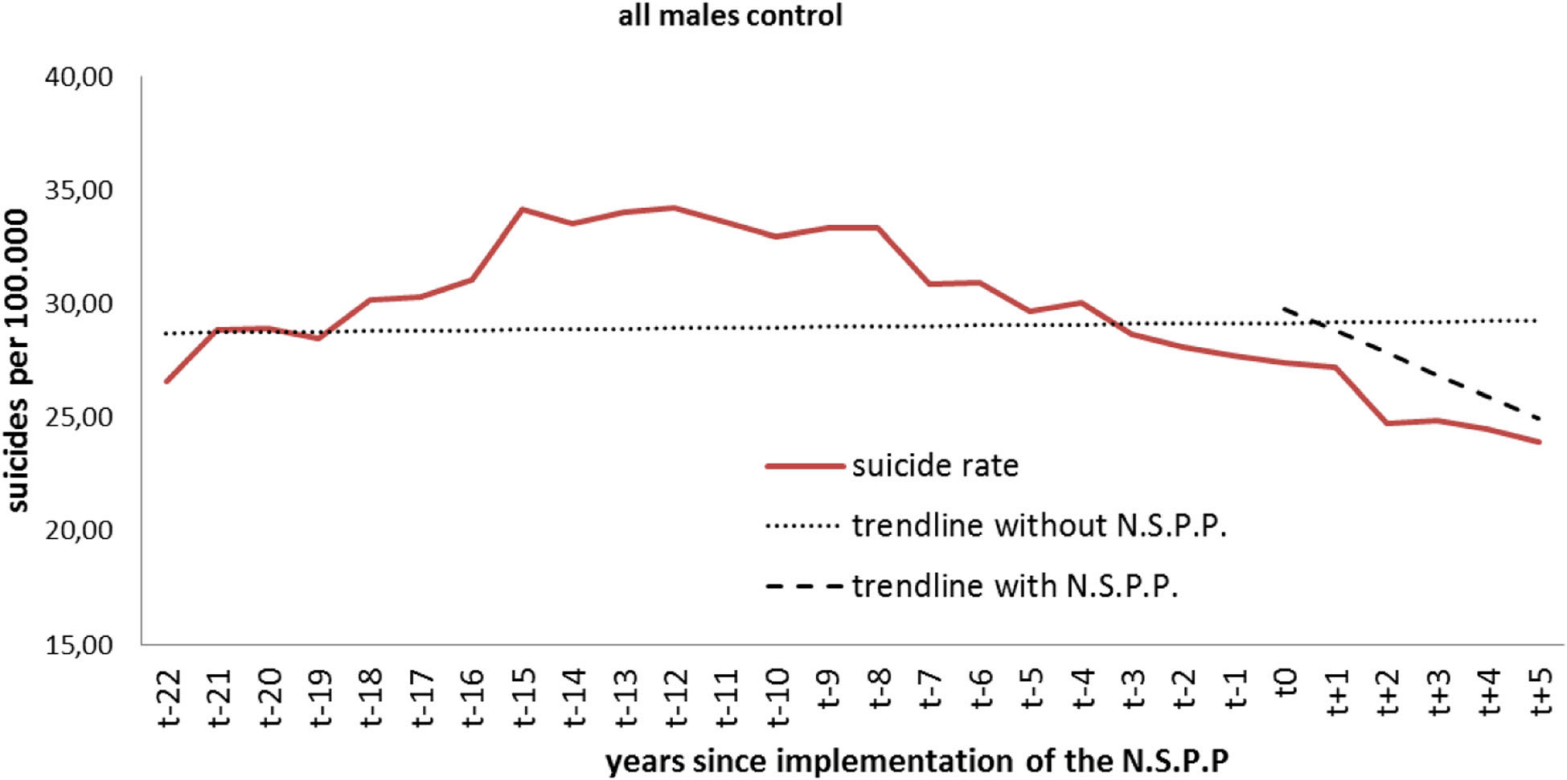
Level change in all control-group males

The average number of suicides per 100.000 for all males in the control countries right at the beginning of the observation period was 28 per year. As we expected, there were no level changes or a significant trend after the period of NSPP implementation in the verum countries. However, although the trend change was not significant, it became clearly negative (Table 3, Fig. 3).

**Table 3:** Control countries: level and trend change in all males

All control males	Coefficient	Standard error	T-statistic	*p*-value
intercept β0	28,703	3051	9408	< 0.001
baseline trend β1	0,020	0,216	0,094	0.926
level change after NSPP β2	0,576	1408	0,409	0.686
effect after 1 year	−0,402	1361	− 0,295	0.770
effect after 2 years	− 1378	1708	− 0,807	0.428
effect after 3 years	− 2351	2276	− 1033	0.312
effect after 4 years	− 3317	2939	− 1129	0.271
effect after 5 years	− 4320	3642	− 1186	0.248
trend change after NSPP β3	−0,972	0,774	− 1257	0.221

The analysis of males according to age group showed no significant trend or level changes in either males < 24 years and those aged 25-to-44 years. We noted a significant (*p* = 0.010) baseline trend of − 0.56 within the 45-to-64 age group, which changed by − 1.1 after the time of NSPP implementation in the verum countries. However, this change was not significant (*p* = 0.303). Interestingly, males older than 65 years showed significant level changes after 2 (*p* = 0.040), 3 (*p* = 0.005), 4 (*p* = 0.002) and 5 (*p* = 0.002) years. We also detected a significant trend change of − 2.6 (*p* = 0.045).

### Analysis of control countries - females

**Fig. 4:**
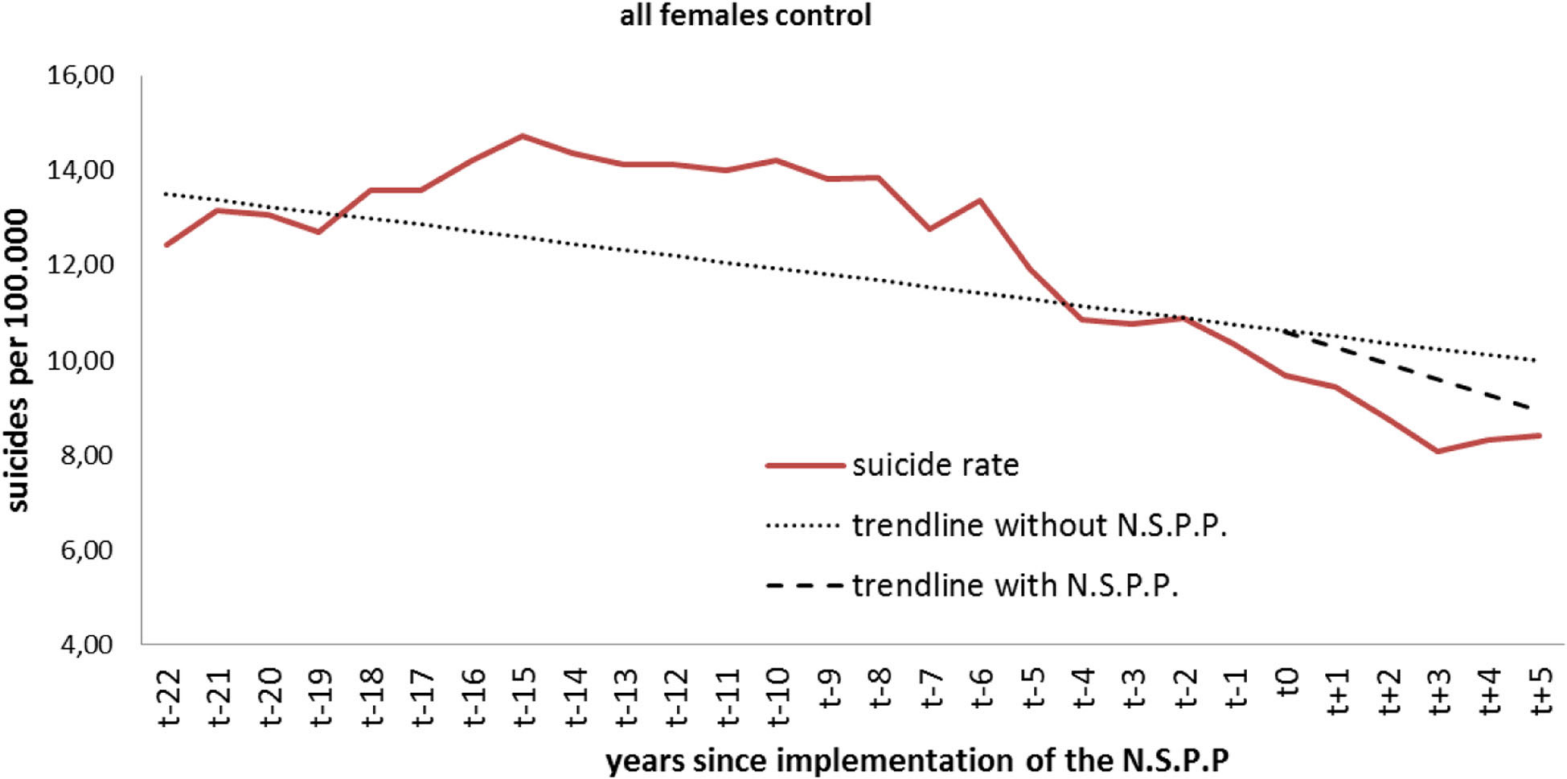
Level change in all control-group females

The analysis of all females in the control countries revealed no significant level or trend changes (Table 4, Fig. 4). The average number of suicides per 100.000 at the beginning of the observation was 13 per year.

**Table 4:** Control countries: level and trend change in all females

All control females	Coefficient	Standard error	T-statistic	*p*-value
intercept β0	13,643	1490	9159	< 0.001
baseline trend β1	−0,131	0,110	− 1200	0.242
level change after NSPP β2	−0,016	0,700	−0,022	0.982
effect after 1 year	−0,226	0,691	−0,328	0.746
effect after 2 years	−0,442	0,888	−0,497	0.624
effect after 3 years	−0,652	1194	−0,546	0.590
effect after 4 years	−0,863	1546	−0,559	0.582
effect after 5 years	− 1084	1916	− 0,566	0.577
trend change after NSPP β3	−0,208	0,404	−0,514	0.612

The analysis of females according to age group showed no changes in trend or level for females < 24 years nor in females in the group of 25-to-44 year-olds. We observed a significant (*p* = 0.001) baseline trend of − 0.4 in the 45-to-64-year-old group. Similar to males, females older than 65 years showed significant level changes in suicide rates after 2 (*p* = 0.020), 3 (*p* = 0.012), 4 (*p* = 0.015) and 5 (*p* = 0.025) years.

### Comparison between verum and control countries

To compare verum and control countries we calculated the difference in the two suicide rates for every year (i.e., the rate of the verum countries minus the rate of the control countries), thus we could analyze both rates in one ARIMA model. Taking the difference collapses the two time series into one and estimates a difference-in-differences effect enables us to make a statement about how the change in the verum countries differed from that in the control countries.

One would expect a statistically significant negative level change a few years after implementation of the NSPP (e.g., the suicide rate in the verum countries would be expected to drop while that in the control countries would remain constant). However, we detected no significant level or trend change regarding the overall rates for all demographic groups including males or females and made the same observation when analyzing the males and females divided into different age groups.

However, the difference in suicide rates right at the beginning of the observation period was significant for all male and female groups except the males ≤24, males aged 25-to-44 years, and females ≤24 (e.g., all males: difference of − 5.6, *p* = 0.018; all females: difference of − 5.2, *p* < 0.001). As mentioned above, segmented regression analysis controls for baseline level and trend.

## Discussion

Overall, this study demonstrates that National Suicide Prevention Programs are effective, but this effect seems to correlate with age and sex.

Segmented regression analyses of interrupted time series data have shown a statistical significant decline in suicide rates in the verum countries in males, with the strongest effects in groups aged 25-to-44 years and 45-to-64 years. We noted a significant effect in females aged 45-to-64 and > 65 years, although this effect was not as strong as it had been in males. We did not detect this effect in the control countries (except in those > 65 years of age). After analyzing the differences in suicide rates between verum and control countries, no significant level changes or trend changes appeared.

Several working groups have investigated various suicide prevention strategies or programs.

Major efforts have focused on the accessibility of suicide means. There is strong evidence that restricting the availability of methods (e.g., firearms) can reduce suicides [22–24]. Men are more likely to use guns as suicide method. That might partly explain the significant effects observed in males age 25-64 years. Another example is the detoxification of the English gas in the 60^s^ which lead to clearly reduced suicide rates [25]. Similar results could be found in Saxony (Germany, “coal gas story”). A 74% reduction in suicide rates were shown due to the detoxification of the city gas [26].

The establishment of suicide prevention centers like the “Samaritans”, “Befrienders International” or “Lifeline” caused a perceptible but nevertheless minor preventive effect [27, 28].

Further approaches like “Tele-Help” or “Tele-Check” were associated with lower suicide numbers [29].

Others have examined the influence of medication on suicidal behavior. Lithium, a mood stabilizer, is well established as a drug that reduces suicides [30].

Advanced training for general practitioners was implemented in the 1980s by the Swedish government. Since general physicians became better able to detect depression than beforehand, suicide rates dropped considerably [31].

The interpretation of statistical data and the causal combination with events or the course of suicide statistics give rise to a complex challenge. Multifarious, unforeseeable factors can play an important role in the appearance of suicidal behavior. Thus the genuine situation in different nations can only be compared under certain limitations.

There are relatively few studies investigating the effectiveness of suicide prevention programs, and those reveal inconsistent outcomes [32–34]. Countries such as Finland and Scotland have reported a significant reduction in suicide rates [35], whereas others (e.g., Norway, Sweden or Australia) reported limited effects in certain subgroups.

Our study results endorse the overall effectiveness of National Suicide Prevention Programs. A major reduction in suicide rates, especially in males over 25 years, is presumably related to all arrangements regarding preventing strategies of these programs rather than to one single strategy. There are a couple of hypotheses as to why we found no statistical differences when comparing verum and control countries:

About 800.000 suicides occurred worldwide representing an annual age-standardized suicide rate of 11.4 per 100,000 population. We know that suicide rates are higher in males (15.0/100000) than in females (8.0/100000). It is acknowledged that three times as many men died by suicide as women; another possible explanation that this study could only reveal differences within the group of men.

Suicide rates are highest in both males and females aged over 70 years. But several countries have different statistical patterns in their age related suicide rates. As the WHO report stated in some countries there is a peak in suicide rates in young adults that subsides in middle age and in other regions suicide rates increase steadily with age [2]. One could argue that our findings in age group 25–64 are partly related to such different patterns.

Prevention programs aiming to help special age groups may play an important role. Within this study’s framework, we were not in a position to analyze other factors associated with changing suicide rates, such as access to and availability of health care providers. Furthermore, the observation period after NSPP implementation was quite short (five years). Certain strategies might well need longer to reveal their effectiveness.

Despite the effort to decrease suicide rates via different approaches also the economic effects are remarkable. Vasiliadis et al. recently showed that suicide prevention programs such as the European Nuremberg Alliance against Depression (NAD) are cost-effective and may result in significant potential cost-savings due to averted suicide deaths and fewer life years lost [36].

It is extremely challenging to investigate changes in implemented prevention strategies such as suicide rates within different countries. Matsubayashi and Ueda (2011) investigated the effect of national suicide prevention programs on suicide rates in 21 OECD nations [37]. Overall, they found that suicide rates decreased after the government initiated a nationwide suicide prevention program, as we did in this study; more so in men than in women. Remarkably, they detected the strongest effects in youth (< 24 years old) and the elderly (> 65 years old). They also noted a limited effect on the working-age population. They discuss those differences as a result of specific goals within the prevention programs, such as reducing the access to firearms. One could argue that a comparison of 21 countries may be too ambiguous, as major cultural, religious, socio-economic and political differences can play an important role. That is why we carefully selected countries that were fairly similar in those specific areas - a clear strength of this study.

A very recent narrative analysis conducted by Zalsmann et al. [38] investigated the effectiveness of different suicide prevention strategies. Due to the heterogeneity of populations and methodology, formal meta-analyses could not be applied. They investigated different suicide prevention methods including school-based awareness program that reduced suicide attempts. They concluded that no one strategy is clearly superior to the others. Our results also support the idea that different approaches appear effective in different groups according to age and gender, for example. That might be another reason for the results found in this study.

Several limitations of this study provide guidance for future research:

- Extensive programs have not been running long enough.
- The present study covered just four control and four verum countries, meaning that our results cannot be extrapolated to other countries.
- The length of our observational period after NSPP implementation is relatively short – later influences could not be excluded.
- Our study approach did not enable us to investigate whether specific components of an NSPP exert different influences on suicide rates.
- Our data did not provide information on whether other activities not implemented in a national strategy such as general welfare programs may also influence suicide rates. According to the WHO report [2], current data show a decrease in suicide rates in different countries even in those without an NSPP, which makes our findings not generalizable.

Despite the encouraging drop in suicide rates, it is very important that future evaluations of suicide prevention programs include the number of suicide prevention interventions implemented successfully as well as the number of hospitalized suicide attempts. The systematic collection of specific data (including suicides and suicide attempts) is key. There are many countries that collect no such data at all or only very minimal data.

## Conclusion

To the best of our knowledge, this is the first study investigating the effectiveness of national suicide prevention programs applying segmented regression analysis of interrupted time series.

Our study implies that the implementation of a national strategy is an effective tool to reduce suicide rates. Special attention should be drawn to different approaches regarding age groups as well concerning females. Future research should investigate longer time periods and different aspects of prevention programs and what other factors may influence suicide rates.

As stated in the WHO’s framework “Public Health Action for the Prevention of Suicide” [2], it is “imperative that governments – through their health, social and other relevant sectors – invest human and financial resources in suicide prevention.”

## Abbreviations

ARIMA model: autoregressive integrated moving average
IASP: International Association for Suicide Prevention
NAD: Nuremberg Alliance against Depression
NSPP: National Suicide Prevention Programs
NYSPS: National Youth Suicide Prevention Strategy
OECD: Organisation for Economic Co-operation and Development
WHO: World Health Organisation
